# Endosomal MR1 Trafficking Plays a Key Role in Presentation of *Mycobacterium tuberculosis* Ligands to MAIT Cells

**DOI:** 10.1371/journal.ppat.1005524

**Published:** 2016-03-31

**Authors:** Melanie J. Harriff, Elham Karamooz, Ansen Burr, Wilmon F. Grant, Elizabeth T. Canfield, Michelle L. Sorensen, Luis F. Moita, David M. Lewinsohn

**Affiliations:** 1 Portland VA Medical Center, Portland, Oregon, United States of America; 2 Pulmonary & Critical Care Medicine, Oregon Health & Science University, Portland, Oregon, United States of America; 3 Instituto Gulbenkian de Ciência, Oeiras, Portugal; 4 Molecular Microbiology and Immunology, Oregon Health & Science University, Portland, Oregon, United States of America; New Jersey Medical School, UNITED STATES

## Abstract

Mucosal-Associated Invariant T (MAIT) cells, present in high frequency in airway and other mucosal tissues, have Th1 effector capacity positioning them to play a critical role in the early immune response to intracellular pathogens, including *Mycobacterium tuberculosis* (Mtb). MR1 is a highly conserved Class I-like molecule that presents vitamin B metabolites to MAIT cells. The mechanisms for loading these ubiquitous small molecules are likely to be tightly regulated to prevent inappropriate MAIT cell activation. To define the intracellular localization of MR1, we analyzed the distribution of an MR1-GFP fusion protein in antigen presenting cells. We found that MR1 localized to endosomes and was translocated to the cell surface upon addition of 6-formyl pterin (6-FP). To understand the mechanisms by which MR1 antigens are presented, we used a lentiviral shRNA screen to identify trafficking molecules that are required for the presentation of Mtb antigen to HLA-diverse T cells. We identified Stx18, VAMP4, and Rab6 as trafficking molecules regulating MR1-dependent MAIT cell recognition of Mtb-infected cells. Stx18 but not VAMP4 or Rab6 knockdown also resulted in decreased 6-FP-dependent surface translocation of MR1 suggesting distinct pathways for loading of exogenous ligands and intracellular mycobacterially-derived ligands. We postulate that endosome-mediated trafficking of MR1 allows for selective sampling of the intracellular environment.

## Author Summary

Tuberculosis, caused by the bacterium *Mycobacterium tuberculosis* (Mtb), remains a global health concern, with an estimated 9 million new cases and 1.5 million deaths each year. Mucosal-associated invariant T (MAIT) cells were recently identified as a non-classical CD8+ T cell subset that responds to intracellular infection with Mtb and other microbes. MAIT cells recognize vitamin B metabolites presented on the Class I like molecule, MR1. MAIT cell recognition is likely to be tightly regulated to allow for the detection of intracellular infection while avoiding tissue damage. In this manuscript, we have characterized the intracellular localization and trafficking of MR1 at basal conditions and in the presence of a known ligand. Furthermore, we have investigated the role of intracellular trafficking in MR1 presentation of ligands to MAIT cells in the context of intracellular infection with Mtb. We show that, in contrast to other Class I molecules, MR1 resides in endosomal compartments and translocates to the cell surface in the presence of ligand. We went on to identify trafficking molecules that are required for the presentation of Mtb antigen to HLA-diverse T cells, and found at least seven trafficking molecules that are specifically involved in regulating MR1-dependent recognition of human MAIT cells. Among these, we show that Syntaxin 18 (Stx18), vesicle-associated membrane protein 4 (VAMP4), and Rab6 play distinct roles in the trafficking of MR1^+^ endosomal compartments. Furthermore, our results demonstrate that exogenously added ligands and those derived during intracellular infection are presented through different mechanisms. Our results provide a conceptual framework underlying the regulation of MAIT cell activation.

## Introduction

Mucosal-Associated Invariant T (MAIT) cells are a class of CD8^+^ T cells that are unique in their use of a semi-invariant TCR, restriction by the highly conserved major histocompatibility complex, class I-related protein 1 (MR1), and their recognition of small molecule metabolites. In support of a role for these cells in host-defense to mucosal infection, human MAIT cells are present in high numbers in mucosal tissues and blood [1, 2] and secrete pro-inflammatory molecules including TNFα and IFN-γ [3, 4]. MAIT cells have the capacity to respond to intracellular pathogens such as *Mycobacterium tuberculosis* (Mtb) [3] and *Francisella tularensis* [5] and animal models demonstrate a requirement for MR1, and by inference, MAIT cells in early control of certain pathogens [5–7]. Although MR1 is ubiquitously expressed in all mammalian tissues examined, surface expression is very low or undetectable in both phagocytic professional antigen presenting cells and non-hematopoetic cells [8, 9]. Previous work demonstrated that MR1 surface expression in mouse cells overexpressing MR1 requires both the MHC-II chaperone, invariant chain (Ii), and trafficking through late endosomal compartments [10]. This is in contrast to the constitutive surface expression of other Class I molecules, and may be key to understanding the regulation of MR1-restricted MAIT cell activation.

The McCluskey and Rossjohn groups have identified Vitamin B metabolites as ligands that bind and stabilize MR1 [11, 12]. With regard to MAIT cell activation, pterins, which are derived from folic acid (6-formylpterin (6-FP and ac-6-FP)), are antagonistic, while the bacterially-derived riboflavin metabolites known as lumazines (RL-6,7-diMe, RL-6-Me-7-OH, and rRL-6-CH_2_OH) are agonists [11]. More recently an additional class of ligands, pyrimidines (5-OE-RU, 5-OP-RU), were identified as potent activators of MAIT cells. These pyrimidines are generated in a chemical reaction between bacterially-derived riboflavin precursor molecules and small metabolites derived from either the host or pathogen [13]. Currently, little is known about the intracellular localization of MR1, how and where MR1 is loaded with these ligands, or the mechanism for MR1 translocation to the cell surface. The ubiquitous expression of MR1 in many tissues, the high frequency of MAIT cells in both the blood and mucosal tissues, and the prevalence of potentially activating ligands likely requires that MR1 loading and trafficking be tightly regulated to prevent indiscriminant MAIT cell activation. Although Ii was proposed as a required chaperone for MR1 [10], we have demonstrated that airway epithelial cells (AEC) are highly efficient at presenting antigen to MAIT cells [14] even though they do not express MHC class II or Ii. The ability of AEC to efficiently present mycobacterial antigens and their proximity to MAIT cells in the lung suggests they may play an important role in early recognition of exposure to Mtb or other pathogens. In this study, we sought to define intracellular trafficking pathways necessary for the presentation of Mtb-derived MR1 ligands by AEC to human MAIT cells.

## Results

### MR1 localizes to vesicular compartments with late endosome and lysosome markers

**Fig 1:**
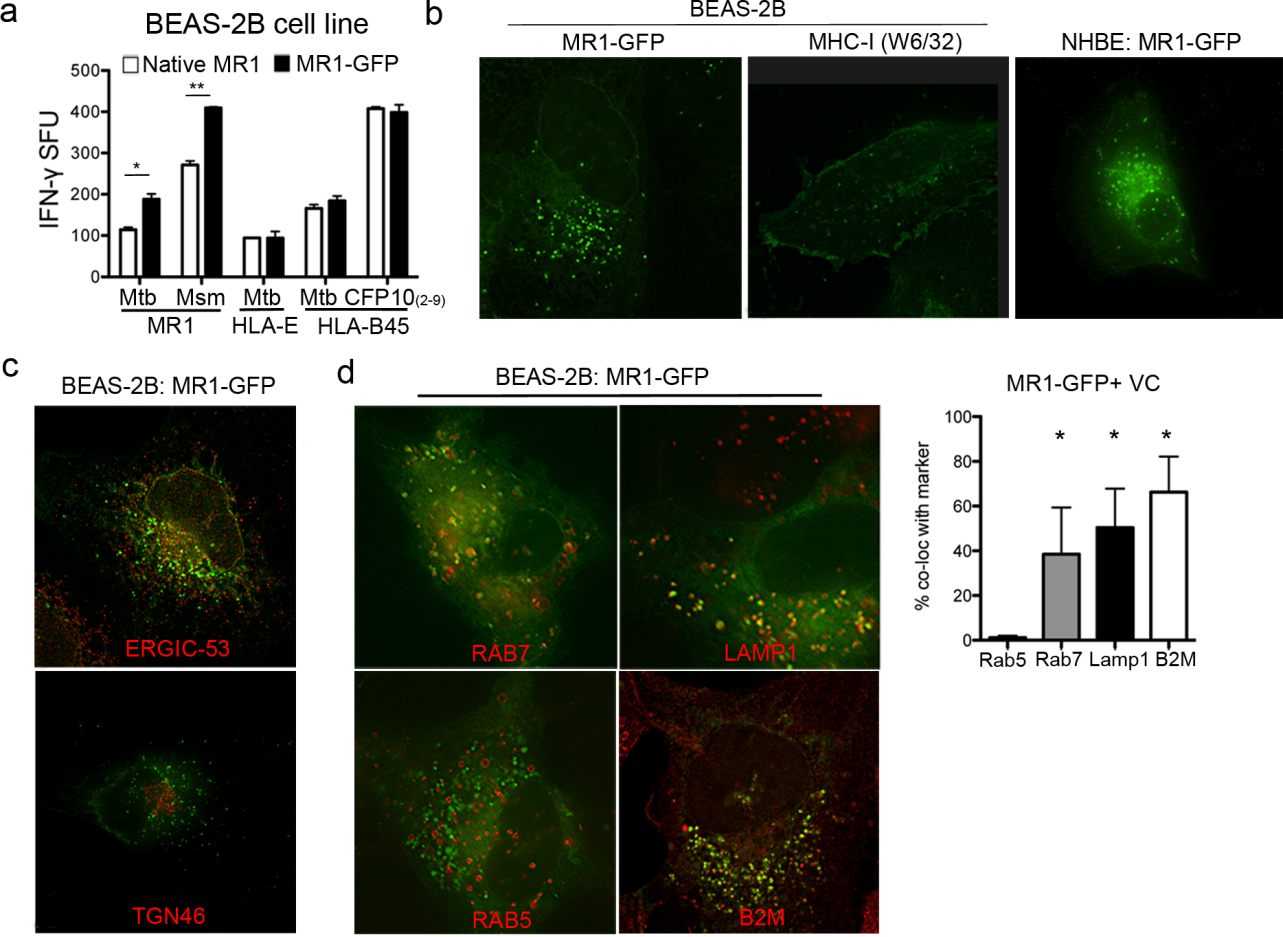
Expression of MR1 in epithelial cells. a) BEAS-2B cells were transfected with pCI:MR1-GFP, infected with Mtb or *M*. *smegmatis* and used as antigen presenting cells in an IFNγ ELISPOT assay with IFNγ production by MR1-, HLA-E, and HLA-B45-restricted T cell clones as a readout for antigen presentation. Error bars represent technical replicates. Data are representative of three independent experiments, *p = 0.03; **p<0.01. b) BEAS-2B or primary NHBE cells were transfected with pCI:MR1-GFP and imaged live after 48 hours. For MHC-I visualization, BEAS-2B cells were fixed and stained with antibody against MHC-I (W6/32). Images are representative of at least three independent experiments. c) BEAS-2B cells were transfected with pCI:MR1-GFP then fixed and stained with α-ERGIC-53 or α-TGN46, and imaged. Images are representative of three independent experiments. d) BEAS-2B cells were transfected with pCI:MR1-GFP then incubated with the CellLights reagents for late endosomes (Rab7), lysosomes (Lamp1), or early endosomes (Rab5) for 48 hours, then imaged live. For β2M, MR1-GFP expressing cells were fixed then stained with α-β2M and imaged. Endosomal compartments were identified and quantified as described in the Materials and Methods. Shown are representative images and the mean and standard deviation of the percent of MR1-GFP^+^ EC co-localizing with the indicated marker from three independent experiments. *p<0.01, compared to Rab5 co-localization.

To determine the basal intracellular localization of MR1 in AEC, we expressed MR1 fused to green fluorescent protein (GFP) in the BEAS-2B airway epithelial cell line and primary normal human bronchial epithelial cells (NHBE). To establish that the MR1-GFP protein was functional, cells expressing MR1-GFP were infected with Mtb or *Mycobacterium smegmatis* and used as antigen presenting cells (APC) in an IFNγ ELISPOT assay. The response of MR1-restricted T cells was increased with MR1-GFP over-expression with no impact on IFNγ production by HLA-E or HLA-B45 restricted T cells (Fig 1A). We next sought to define the subcellular localization of MR1-GFP using high-resolution deconvolution microscopy. As expected, in both BEAS-2B and NHBE cells, the majority of MR1 observed was intracellular, with expression in an endoplasmic reticulum (ER)-like subcellular compartment as well as endosomal compartments (EC) (Fig 1B, S1 Movie). This was in contrast to MHC-I, which was largely localized to the plasma membrane (Fig 1B). Staining with antibodies against the ER-Golgi intermediate compartment (ERGIC-53) the trans-Golgi network (TGN-46) demonstrated that a proportion of the MR1-GFP localized to these compartments (Fig 1C), while subsets of the MR1-GFP^+^ EC co-localized with markers that define late endosomes or lysosomes (Rab7, Lamp1) but not early endosomes (Rab5) (Fig 1D, S2 Movie). The majority of the MR1-GFP^+^ EC also co-localized with β-2-microglobulin (β-2M) (Fig 1D). These MR1^+^ β-2M^+^ EC have a similar phenotype to the Mtb compartment in AEC [14] and may represent a pre-synthesized pool of MR1 that could be loaded in the context of intracellular infection.

### Incubation with 6-FP results in translocation of MR1 to the cell surface

**Fig 2:**
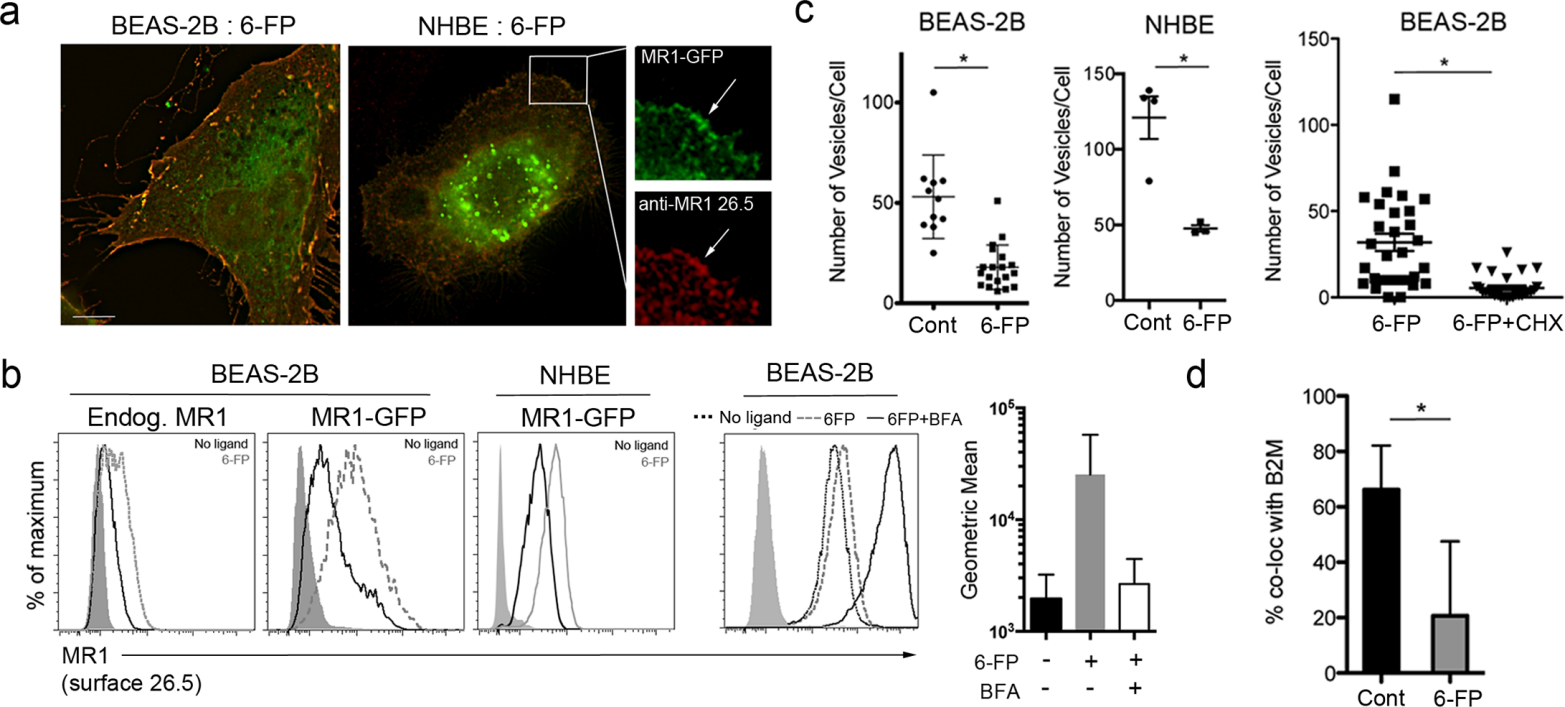
Cell surface translocation of MR1 in the presence of ligand. a) BEAS-2B or primary NHBE cells were transfected with pCI:MR1-GFP and incubated for 30 hours. Transfected cells were then incubated with 6-FP for 18 hours. For imaging, cells were fixed and surface stained with α-MR1 (26.5), then imaged. In NHBE cells, MR1 surface staining (red) in MR1-GFP-expressing cells (green) is denoted by the arrows in the enlarged insets. Images shown are representative of at least three independent experiments. b) BEAS-2B or NHBE cells were treated as described in a). Following 6-FP treatment cells were harvested and surface stained on ice with α-MR1 (26.5). After staining, cells were fixed and analyzed by flow cytometry. Data shown are representative of at least three independent experiments. Where indicated, cells were treated with 100ng/ml brefeldin A (BFA) for 2 hours prior to 6-FP addition. Shown is a representative histogram demonstrating BFA blockade of 6-FP-dependent surface stabilization and the geometric mean and SEM of from three independent experiments. c) BEAS-2B or NHBE cells were transfected with pCI:MR1-GFP and treated with 6-FP as described above. Where indicated, cells were treated with10ug/ml cycloheximide (CHX) for 2 hours prior to 6-FP addition. Cells were fixed, and where indicated, stained with α-β2M, and imaged. Shown are results from one of at least three independent experiments. The Mann-Whitney test was used to determine the statistical significance of CHX treatment. For all other statistical comparisons, a Student’s t-test was used, *p<0.01. d) MR1-GFP^+^ β2M^+^ EC were identified and quantified as described in the Materials and Methods. Each dot represents the number of endosomes in one cell for any of the conditions. Shown are results from one of at least three independent experiments, *p<0.01.

Recent reports indicate that incubation of cells with acetyl-6-FP results in cell surface stabilization of MR1 [12]. To define the impact of exogenously added ligand on the intracellular localization of MR1 in BEAS-2B and primary NHBE cells, MR1-GFP expressing cells were incubated with 6-FP. As expected, incubation with 6-FP resulted in translocation of MR1-GFP to the cell surface in both BEAS-2B and NHBE cells, detected by surface staining with the α-MR1 26.5 antibody (red) (Fig 2A), which recognizes folded MR1 [13, 15]. Flow cytometry confirmed 6-FP dependent cell-surface translocation in cells expressing MR1-GFP and native MR1 (Fig 2B). Inhibiting ER-Golgi transport with brefeldin A (BFA) dramatically decreased the 6-FP-dependent cell surface stabilization of MR1 (Fig 2B), indicating that the dominant processing and presentation pathway for 6-FP requires ER-Golgi transport.

The 6-FP-dependent translocation of MR1 to the cell surface was accompanied by a decrease in the number of MR1-GFP^+^ EC in BEAS-2B and primary NHBE cells (Fig 2C) and MR1-GFP+ EC were further depleted by cycloheximide (CHX) treatment (Fig 2C). When we analyzed the co-localization of β-2M with the MR1-GFP^+^ EC remaining following treatment with 6-FP, we found that the percentage of MR1-GFP^+^ β-2M^+^ EC decreased (Fig 2D). Together these data indicate that the MRI^+^ β-2M^+^ EC are a source of intracellular MR1 molecules available for ligand-binding and translocation to the cell surface.

### Identification of trafficking molecules in AEC required for presentation of Mtb ligands on MR1

**Fig 3:**
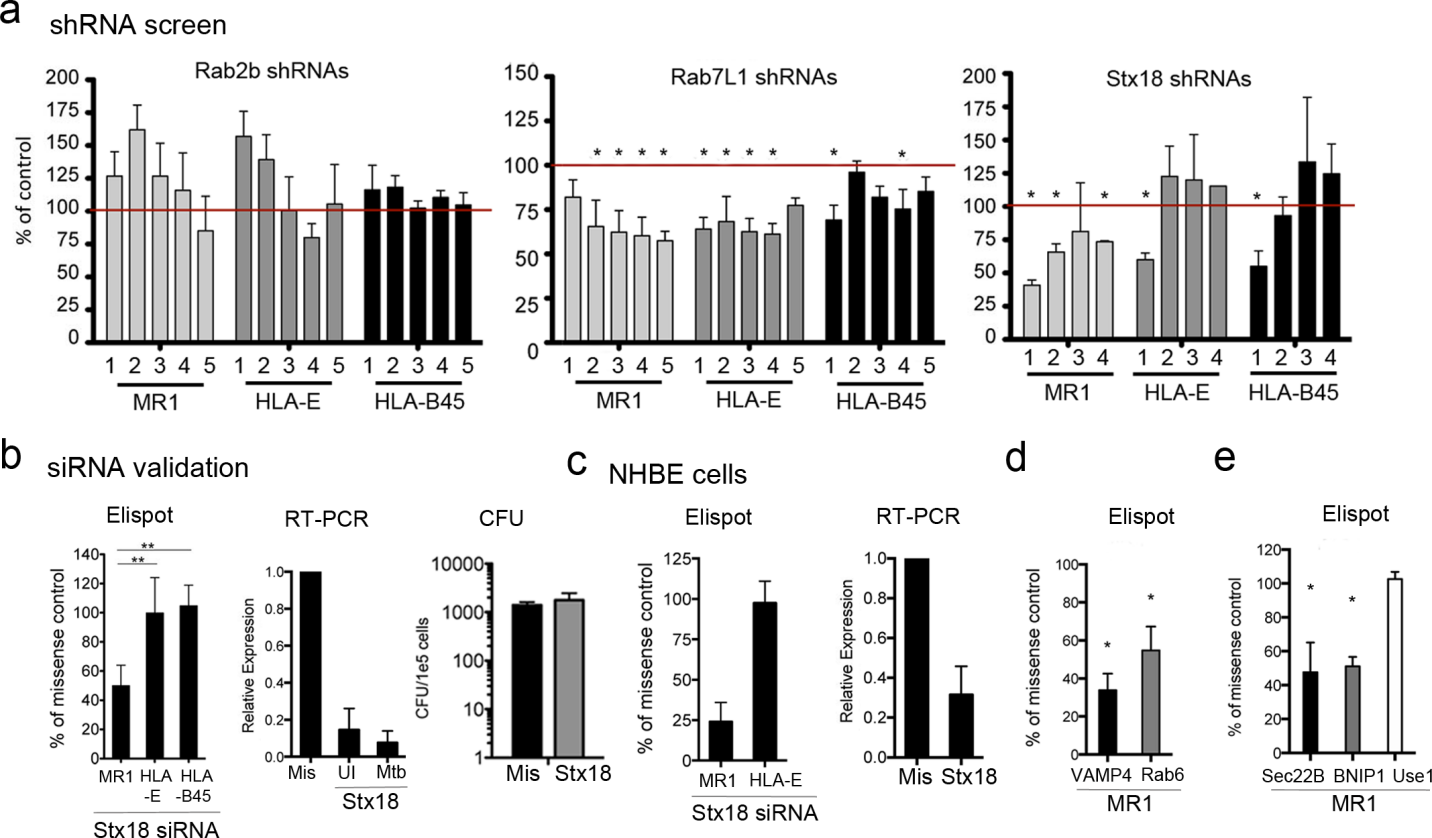
Identification of trafficking molecules involved in presentation of Mtb ligands on MR1. a) A lentiviral shRNA library of trafficking molecules was screened by IFNγ ELISPOT assay with MR1, HLA-E, and HLA-B45 restricted T cell clones as described. Each bar represents separate shRNA wells and each well was analyzed individually in relation to the mean and characterized as a hit if it was at least 25% below the mean. The mean was normalized to 100% and is represented by red line. Results from shRNA knockdown of Rab2b, Rab7L1, or Stx18 from three independent experiments are shown. Stars indicate gene specific shRNA wells that met the candidate selection criteria. Candidates were selected if at least 2 of 5 independent shRNAs resulted in reduced T cell response. b) BEAS-2B cells were treated with missense or Stx18 siRNA, infected with Mtb (MOI:5), and used as APC in an IFNγ ELISPOT assay with 1x10^4^ MR1, HLA-E, or HLA-B45 restricted T cells clones per well. Shown are the results using 5 x 10^3^ APC that have been normalized to the response to the Missense siRNA treated cells (100%). **p<0.01. Error bars represent the mean and SEM from four independent experiments. qRT-PCR was performed on cDNA synthesized from mRNA isolated from missense or Stx18 siRNA silenced BEAS-2B cells that were uninfected or infected with Mtb (MOI:5). Error bars represent the mean and SEM for three independent experiments. Mtb colony forming units (CFU) from 1x10^5^ infected cells were determined by lysing and plating serial dilutions on 7H10 agar plates. Error bars represent the mean and SEM from 3 independent experiments. c) NHBE cells were treated with missense or Stx18 siRNA and infected with Mtb (MOI:10). IFNγ ELISPOT and qRT-PCR analyses were performed as described for BEAS-2B cells. Error bars represent the mean and SEM from two independent experiments. d) BEAS-2B cells were treated with missense, VAMP4, or Rab6 siRNA and infected with Mtb (MOI:5). IFNγ ELISPOT and qRT-PCR analyses were performed as described above. Error bars represent the mean and SEM from at least three independent experiments, *p<0.01 compared to missense. e) BEAS-2B cells were treated with missense, Sec22b, BNIP1, or Use1 siRNA and infected with Mtb (MOI:5). IFNγ ELISPOT and qRT-PCR analyses were performed as described above. Error bars represent the mean and SEM from at least two independent experiments, *p<0.01 compared to missense.

The identification of vitamin B metabolites as MR1 ligands [11], the observation that processing and presentation is both TAP and proteasome independent [3, 10], and apparent requirement for ligand in the cell surface stabilization of intracellular MR1 suggest a unique mechanism for MR1 antigen processing and presentation. To define this mechanism in the context of intracellular infection with a pathogen, we designed a screen to identify trafficking proteins that are essential for MR1-dependent MAIT cell activation in the setting of intracellular infection with Mtb (S1 Fig). Here, shRNA knockdown of trafficking molecules in BEAS-2B cells expressing native MR1 was performed, and T-cell dependent release of IFN-γ was measured following Mtb infection. To identify MR1-specific mechanisms, human Mtb-reactive CD8^+^ T cell clones of known specificity (MR1 (D426 B1), HLA-E (D160 1–23), and HLA-B45 (D466 D6)) were tested in parallel with the same Mtb-infected APC. Candidate proteins were identified by the selection criteria described in the Materials and Methods. Briefly, to reduce the impact of non-specific shRNA targeting, genes where at least two independent shRNAs resulted in reduced T cell recognition were considered candidates for subsequent siRNA validation. Based on these criteria, we identified candidate genes that fell into three categories with regard to recognition by MAIT cells: 1) Genes where silencing did not result in reduced recognition by any of the T cell clones; 2) Genes where silencing resulted in reduced recognition by all of the T cell clones; and 3) Genes where silencing resulted in reduced recognition specifically by MAIT cells. Examples are shown in Fig 3A, where silencing of Rab2b with multiple shRNAs in BEAS-2B cells did not inhibit Mtb-dependent IFN-γ release by any of the T cell clones tested, while silencing of Rab7L1 by multiple shRNAs inhibited responses by all T cell clones tested, including MAIT cells. In contrast, silencing of Stx18 with shRNA selectively inhibited only the response of the MAIT cell clone, D426B1, according to the selection criteria (Fig 3A). In total, we identified 27 candidate genes where shRNA knockdown specifically resulted in reduced MAIT cell recognition of Mtb-infected BEAS-2B cells.

### The ER-associated SNARE Stx18, and the endosome-associated trafficking molecules VAMP4 and Rab6 are critical for MR1-dependent MAIT cell activation in the context of Mtb infection

To date, we have evaluated ten of the 27 MAIT cell-specific candidates using siRNA knockdown. Gene knockdown was confirmed using RT-PCR, and T cell-dependent release of IFN-γ was responses were evaluated by ELISPOT assay. Silencing of eight of the ten candidates tested by siRNA resulted in reduced response by the MAIT cell clone, seven of which were confirmed to specifically impact only the MAIT cell clone. All candidates evaluated are listed in Table 1 and representative results are illustrated in Fig 3B. Here, Mtb-infected APC silenced with Stx18 siRNA had a substantially reduced ability to activate the MR1-restricted clone, with no effect on activation of the HLA-E and HLA-B45-restricted clones, validating the results of the shRNA screen. Furthermore, infection and intracellular viability of Mtb were not affected by Stx18 silencing as demonstrated by quantitative culture of intracellular Mtb (Fig 3B). Similarly, Stx18 silencing in Mtb-infected NHBE cells resulted in diminished MR1-dependent recognition of infection with Mtb (Fig 3C). Together, these data suggest that Stx18 is involved in a mechanism that selectively impacts MR1-dependent antigen processing and/or presentation of Mtb antigen by AEC. Because MR1 was observed in intracellular compartments in the endosomal pathway, we also focused on two endosome-associated trafficking proteins, the R-SNARE vesicle-associated membrane protein 4 (VAMP4) and Rab6, a small GTPase protein. Similar to what was observed for Stx18, following VAMP4 or Rab6 silencing with siRNA, there was diminished ability of Mtb-infected APC to activate MR1-restricted T cells (Fig 3D).

**Table 1:** MR1 candidates evaluated using siRNA gene knockdown.

**Gene Name**	**Number of shRNA hits**	**Validated by siRNA**
Bet1	2	No
Rab3a	2	Yes
Rab3c	2	Yes^a^ ^,^ ^b^
Rab3GAP2	2	Yes
Rab6	3	Yes^b^
Rab34	2	Yes
Sec22b	3	Yes^b^
Stx18	3	Yes^b^
Vamp4	3	Yes^c^
Ykt6	2	No

^a^ siRNA knockdown resulted in a similar functional effect (measured by ELISPOT assay) as observed using shRNA, however we were unable to clearly show gene expression in BEAS-2B cells by qRT-PCR

^b^ Tested with multiple independent siRNAs

^c^ siRNA knockdown had a variable impact on response of the HLA-E-restricted T cell clone

### Stx18-dependent Golgi-ER retrograde transport is not required for MR1 processing and presentation of Mtb ligands

Stx18 is an ER-localized soluble NSF attachment protein receptor (SNARE) protein of the Qa-SNARE family best known as a member of a SNARE complex regulating Golgi-ER retrograde transport consisting of Stx18, BNIP1, Use1 (also called p31, D12, or MDS032), and Sec22b [16]. Because Sec22b was also validated from the shRNA screen as an MR1-specific candidate, we analyzed the role of this SNARE complex in Mtb antigen presentation to MAIT cells. SNAREs known to partner with Stx18 were silenced by siRNA and tested for their ability to activate MAIT cells. As expected, knockdown of Sec22b resulted in a similar reduction in MAIT cell activation as was observed for Stx18. Knockdown of BNIP1 also resulted in a reduction in MAIT cell activation, however knockdown of Use1 did not have any impact (Fig 3E). The SNAREs in this complex can also play a role in ER-mediated phagocytosis through interactions with plasma membrane SNAREs [17] as well as post-Golgi trafficking pathways [18]. In fact, Stx18 has unusual properties for a Qa-SNARE, as it has a SNARE motif that is longer than conventional SNAREs [19] and undergoes a different mechanism of SNARE complex assembly compared to other ER SNAREs [20] that may allow it to function independently of this complex. These data suggest MR1 processing and presentation may require a Stx18-mediated trafficking pathway distinct from the previously characterized Golgi-ER retrograde transport pathway.

### Mtb-derived ligands are loaded on MR1 using a pathway distinct from 6-FP

**Fig 4:**
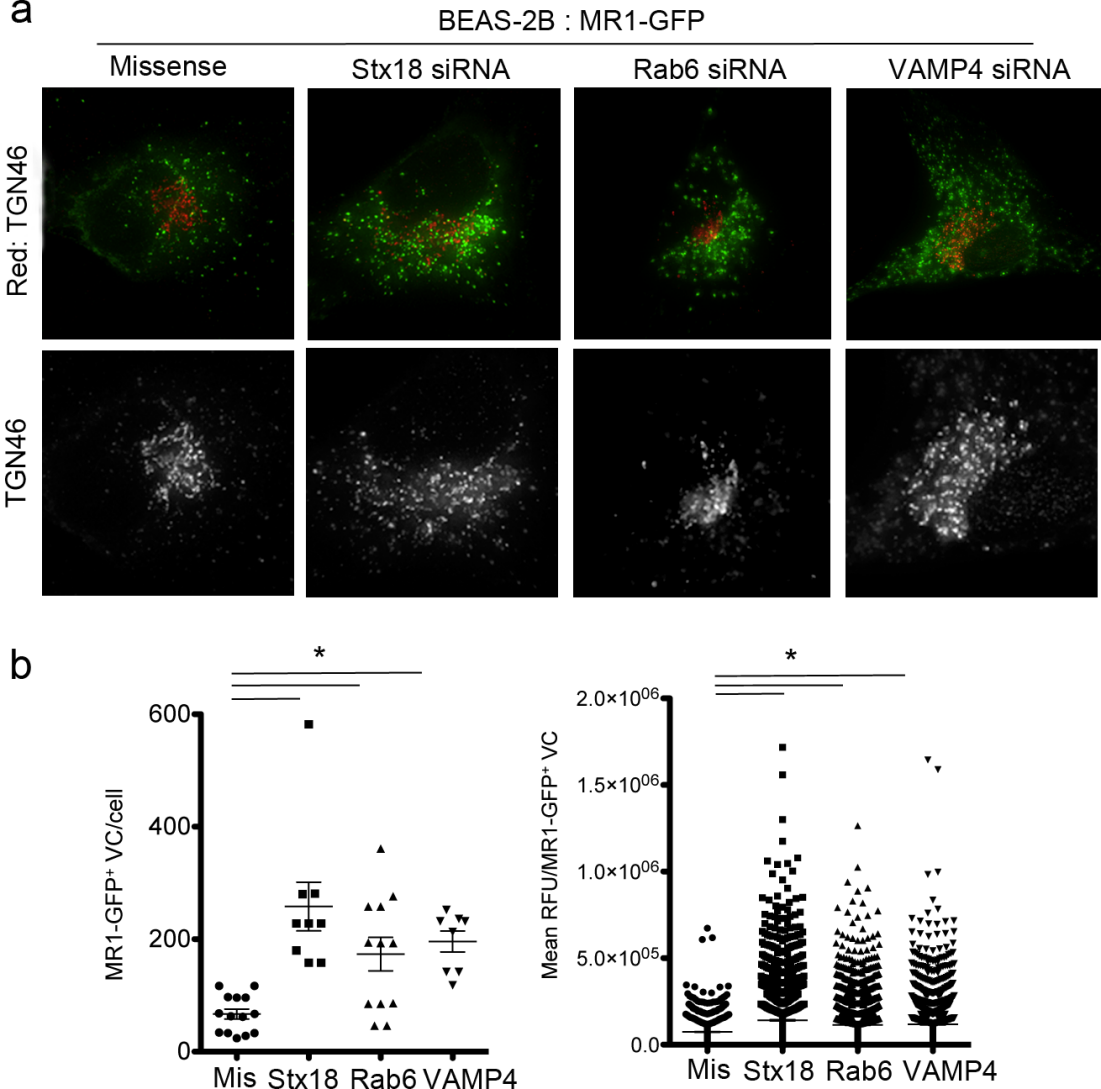
Stx18, VAMP4, and Rab6 silencing alter TGN structure and intracellular MR1 localization. a) BEAS-2B cells treated with missense, Stx18, Rab6, or VAMP4 siRNA were transfected with pCI:MR1-GFP for 48 hours, then fixed and imaged. Shown are representative images of the cells used to enumerate MR1-GFP^+^ EC. TGN46 staining is shown at 1.5x magnification in greyscale below each image to demonstrate dispersion or contraction of the TGN. b) MR1-GFP^+^ EC were identified and quantified as previously described. On the left, each dot represents the number of MR1-GFP^+^ EC in a single cell, and the data shown is representative of 4 independent experiments. On the right, each dot represents the mean intensity of GFP signal in one individual MR1-GFP^+^ EC. The data represent all endosomes from the cells plotted on the left, and are representative of 4 independent experiments. The Mann-Whitney test was used to determine statistical significance, *p<0.01.

To understand the mechanism by which these trafficking molecules regulate processing and presentation of Mtb antigens on MR1, we looked for alteration in MR1 localization following siRNA treatment. In HeLa cells, knockdown of Stx18 has been reported to affect the ER, ER-Golgi intermediate compartment (ERGIC), and Golgi including disruption of the Golgi stacks and ER exit sites and disorganization of ER membranes [21]. Knockdown of VAMP4 in HeLa cells also results in dispersion of the Golgi [22] whereas knockdown of Rab6 does not disperse the Golgi structure, but makes it more compact [23]. To confirm these observations, Stx18, VAMP4, and Rab6 were silenced in BEAS-2B cells. Knockdown of Stx18 and VAMP4 resulted in the dispersion of the Golgi, while the structure of the Golgi in Rab6 silenced cells was more compact (Figs 4A and S2). In the context of MR1-GFP expression, knockdown of Stx18, VAMP4, and Rab6 resulted in an increase in the number of MR1-GFP^+^ EC (Fig 4B) and in the amount of MR1 in those compartments, as measured by the mean intensity of GFP per EC (Fig 4B).

**Fig 5:**
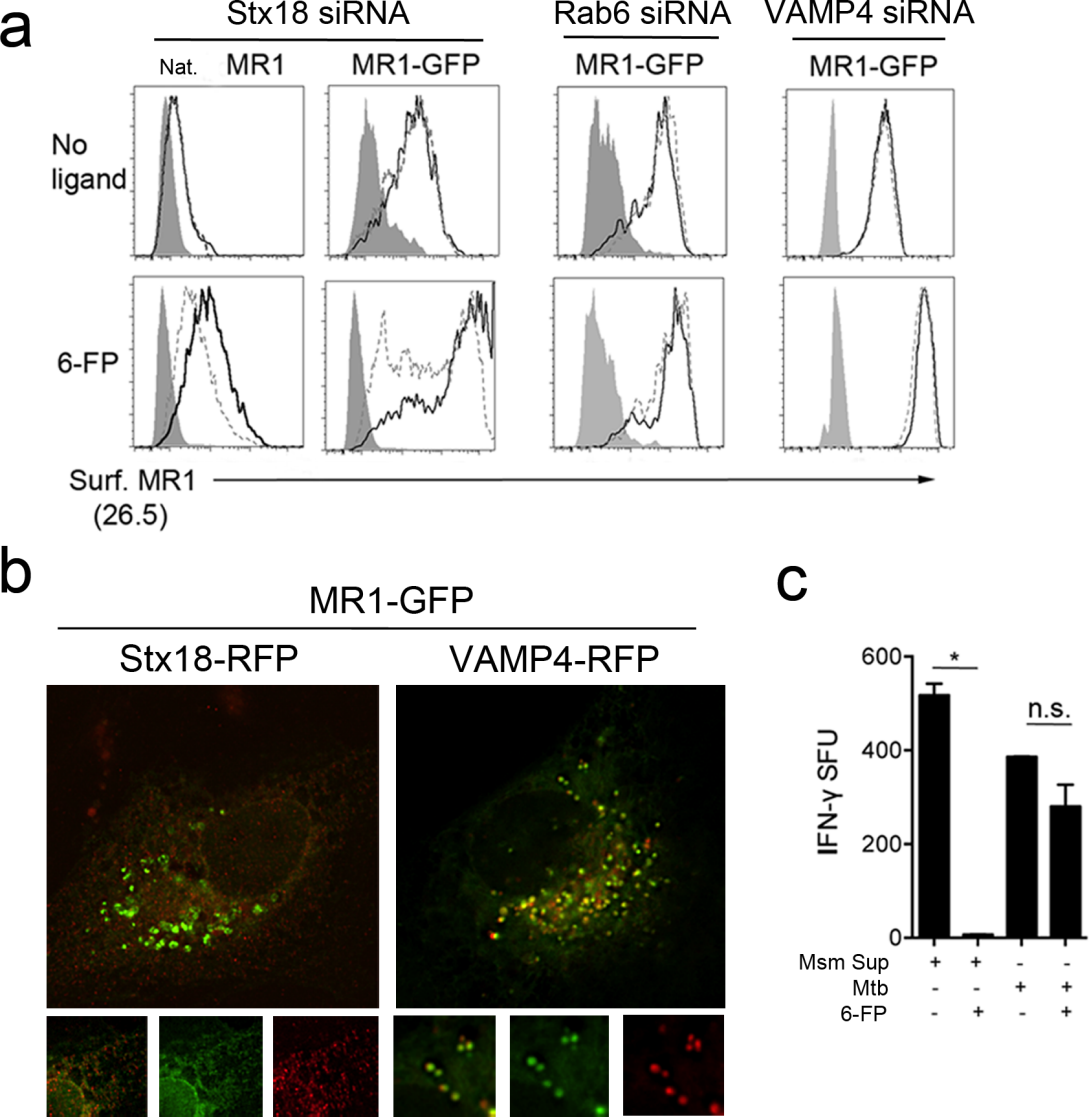
Mtb-derived ligands are loaded on MR1 using a pathway distinct from that of 6-FP. a) BEAS-2B cells treated with missense, Stx18, Rab6 or VAMP4 siRNA were transfected with pCI:MR1-GFP for 30 hours, then incubated or not with 6-FP for 18 hours. Cells were then harvested, surface stained with α-MR1 (26.5) and examined by flow cytometry. Cells were gated on expression of MR1-GFP based on an untransfected control. Shaded histograms represent the isotype control. Black solid histograms represent the cell surface expression of MR1 after treatment with missense control siRNA, while the dashed histograms represent the cell surface expression of MR1 in the after treatment with the indicated siRNA. Data is representative of three independent experiments. b) For Stx18, BEAS-2B cells were transfected with MR1-GFP and incubated for 24 hours, then transfected with Stx18-RFP and incubated for 16 hours. For VAMP4, BEAS-2B cells stably expressing MR1-GFP were transfected with VAMP4-RFP and incubated for 18 hours. Cells co-expressing MR1-GFP and Stx18 or VAMP4 were imaged live. Images are representative of at least three independent experiments. c) BEAS-2B cells were infected with Mtb (MOI:8) for 18 hours or incubated with supernatant from *M*. *smegmatis* cultures (Msm Sup). Where indicated, cells were pretreated with 6-FP for 2 hours prior to infection or addition of supernatant. Cells were then used as APC in an IFNγ ELISPOT assay with IFNγ production by an MR1-restricted T cell clone as a readout for antigen presentation. Data are representative of two independent experiments, *p<0.01, n.s. not significant.

The functional defect observed by ELISPOT and the increase in MR1-GFP+ EC led us to hypothesize that silencing of these genes could result in diminished cell surface expression of MR1 in the presence of a ligand. Knockdown of Stx18, VAMP4, and Rab6 did not alter basal cell surface expression of MR1 in cells expressing endogenous MR1 or over-expressing MR1-GFP (Fig 5A). However, Stx18 was required for ligand-dependent translocation of MR1 to the cell surface as Stx18 knockdown resulted in reduced translocation of MR1 to the cell surface after incubation with 6-FP (Fig 5A). Knockdown of VAMP4 or Rab6 had no impact on translocation of MR1 to the surface with 6-FP incubation (Fig 5A). Stx18, VAMP4, and Rab6 silencing all resulted in similar reduction in MR1-restricted T cell response to Mtb-infected cells. The discordant results with 6-FP dependent MR1 surface stabilization in Stx18- and VAMP4- or Rab6-silenced cells support a model in which the processing and presentation pathways for exogenously added 6-FP and intracellular bacteria are distinct. Consistent with this model, we observed colocalization of Stx18 with MR1 in the ER, while VAMP4 colocalized with MR1 in EC (Fig 5B).

To further distinguish exogenously delivered versus intracellular antigen, we analyzed the impact of pre-treatment with 6-FP, a MAIT cell antagonist, on the ability of MAIT cells to respond to exogenous or endogenous antigen in the context of native MR1. Incubation of APC with *M*. *smegmatis* supernatant resulted in MAIT cell activation, which could be abrogated by pre-incubation of the APCs with 6-FP (Fig 5C). In contrast, in cells infected with Mtb, pre-incubation with 6-FP did not significantly reduce MAIT cell activation (Fig 5C). These data suggest that for processing and presentation of intracellular Mtb-derived antigen, there is an endosomal pool of MR1 that is distinct from the pool preferentially loaded with 6-FP.

## Discussion

MAIT cells are a unique class of MR1-restricted T cells capable of recognizing small molecule vitamin B metabolites. There is a growing appreciation for the importance of these cells in the mucosal response to intracellular microbial infection. At present, little is known about the mechanisms by which this novel class of antigens are processed and presented. As we have demonstrated previously, MAIT cells recognize Mtb-infected dendritic and epithelial cells and are present in high numbers in lung tissues [3, 14, 24]. We postulate that the circumstances by which MAIT cells become activated is tightly regulated to allow for the recognition of intracellular infection while avoiding tissue damage. Here we have demonstrated that MR1 is primarily localized to an endosomal compartment, and have identified trafficking molecules responsible for presentation of Mtb antigens on MR1. These results provide a framework with which to understand the mechanisms regulating activation of MAIT cells.

While we and others have shown that exogenous ligands such as the short-lived ribityllumazine metabolites can be presented in the context of MR1 *in vitro*, the extent to which exogenous ligands contribute to MAIT cell activation *in vivo* is unknown. Our data support the hypothesis that regulation of MAIT cell activation is critically dependent on intracellular infection. Cell-surface localization of classical Class I molecules is dependent on binding by a peptide that can be either endogenous or exogenous. This is in contrast to MR1, which we demonstrate is primarily localized to endosomal compartments with features of late endosomes and lysosomes, consistent with previous findings in mouse embryonic fibroblasts [10]. The difference in intracellular localization between classical Class I molecules and MR1 suggest that translocation to the cell surface is a key event for MAIT cell recognition of intracellular infection. By maintaining a dynamic endosomal store of MR1, ligands derived from intracellular pathogens can be rapidly loaded and presented on the cell surface. MR1 that does not encounter intracellular ligand may be rapidly recycled or degraded, keeping access to ligands from normal extracellular gut flora to a minimum. These findings led us to explore the role of endosomal trafficking in presentation of Mtb ligands on MR1 to MAIT cells.

**Fig 6:**
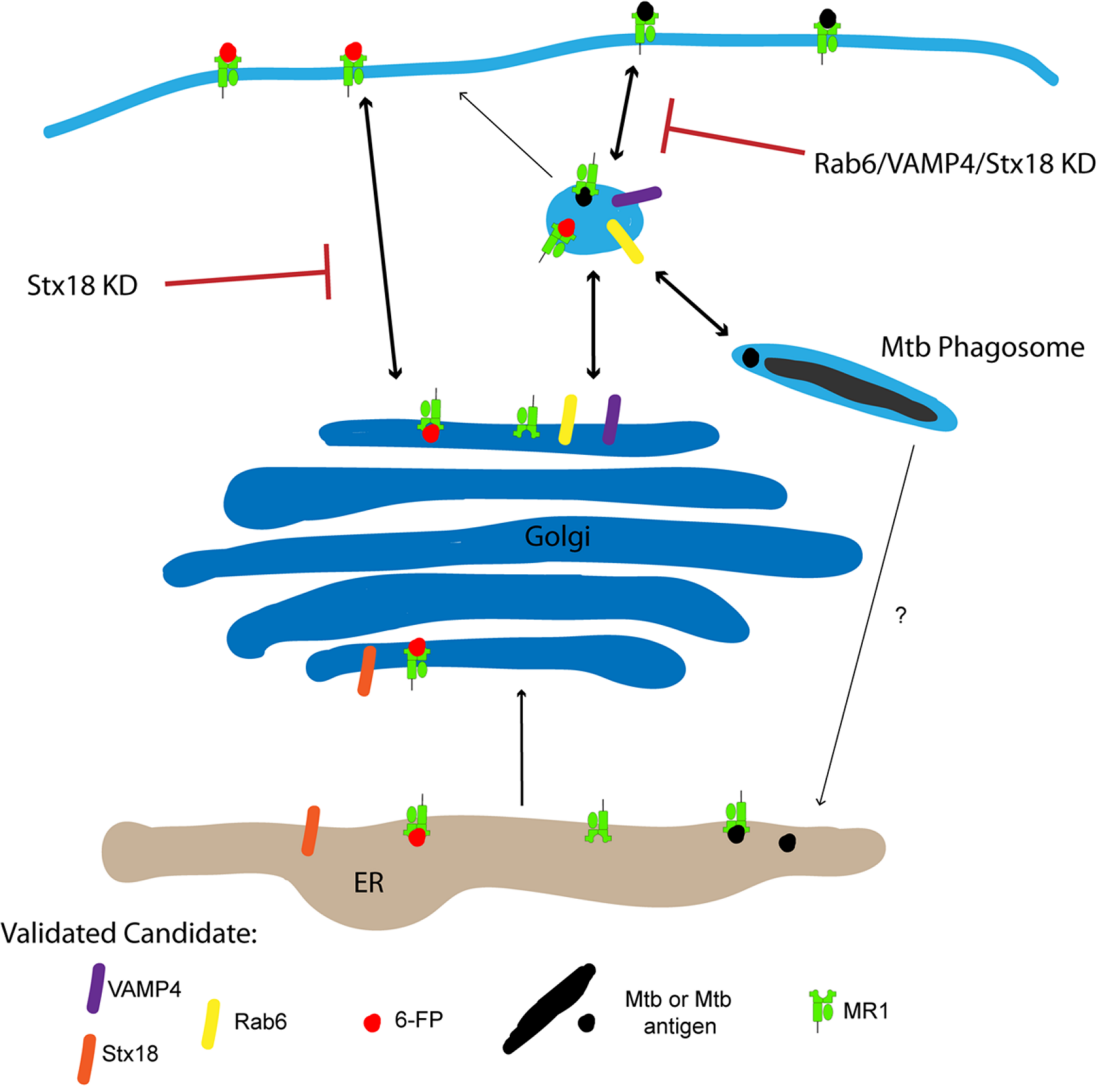
Model of MR1 trafficking. Our data support the hypothesis that the primary MR1 presentation pathway for exogenous ligands like 6-FP is through the ER. MR1 loading of exogenous ligand through this pathway requires the ER-localized SNARE protein, Stx18, but not the TGN-endosome localized trafficking molecules Rab6 and VAMP4. In contrast, the MR1 presentation pathway for ligands derived from intracellular pathogens like Mtb is primarily through endosomal compartments, and requires Stx18, Rab6, and VAMP4.

Using lentiviral shRNA knockdown in Mtb-infected APC, we identified a set of trafficking molecules specifically involved in the presentation of MR1-ligands to MAIT cells. Our results lead us to postulate a model where, in contrast to classical ER-associated Class I antigen processing and presentation, the expression of MR1 in endosomal compartments is critical to regulation of the processing and presentation of MR1 ligands generated in the context of intracellular infection with Mtb. In this model, we seek to explain 1) the proteins involved in generation of MR1^+^ EC, 2) the location where MR1 interacts with ligand, and 3) the ability of loaded MR1 to translocate to the cell surface. While we cannot yet say where MR1 is interacting with Mtb ligand based on our data, we can conclude that MR1 is likely interacting with exogenously added ligands such as 6-FP primarily in a pre-Golgi compartment such as the ER. We have identified proteins, Stx18, VAMP4, and Rab6, which play a role in maintaining the structure of the TGN, and ultimately regulate the translocation of MR1 to the cell surface in the context of infection with Mtb (Fig 6). In the context of our model, 6-FP is loaded through a distinct pathway from those derived in the context of intracellular infection. The ER-associated SNARE, Stx18, appears to play a role in both of these pathways, whereas the TGN-endosome localized Rab6 and VAMP4 uniquely contribute to the loading of Mtb ligands. These data support a role for the MR1^+^ EC in loading of Mtb ligands.

While we have identified a preliminary framework by which these trafficking proteins participate in presentation of Mtb antigens on MR1, there are still many unanswered questions about how MR1 samples the intracellular environment. At present, there are three ways in which the immune system is known to sample the endosomal environment, both of which involve exchange of ligand. The first is through MHC Class II antigen presentation, where peptides derived from the endosomal pathway are exchanged with the invariant chain (Ii) on MHC Class II molecules in a late endosomal compartment known as the MHC Class II compartment (MIIC) (reviewed in [25]). The second is through the MHC Class I endosomal recycling pathway, where MHC Class I molecules are loaded with endosomally-derived peptides through the action of cathepsins and peptide exchange [26]. The third is through CD1 antigen presentation, where lipid antigens can be sampled in early and late endosomes, including the MIIC (reviewed in [27, 28]). Unlike MHC-II, classical MHC-I, and CD1 molecules, neither self-ligands nor chaperones have been defined for MR1. Although invariant chain (Ii) was proposed as a required chaperone for MR1 [10], we showed that BEAS-2B cells are highly efficient at presenting antigen to MAIT cells [14] even though they do not express MHC class II or Ii. Based on this, we hypothesize that there are chaperones in addition to Ii that are able to stabilize MR1 and contribute to endosomal loading of antigen.

Peptide-loaded classical Class I molecules translocate to the cell surface regardless of whether they contain endogenous or exogenous peptides. CD8^+^ T cells sample all peptides that are presented, and the ability of T cells to discriminate self from non-self is determined by the specificity of the T cell receptor (TCR). Here, constitutive cell surface localization of classical Class I molecules does not lead to indiscriminant T cell activation as thymic selection serves to remove T cells that are auto-reactive. For MAIT cells, the TCR appears to have broader reactivity from that of classical CD8^+^ T cells. Instead, recognition by MAIT cells may be dependent on the regulated intracellular loading of ligand that in turn leads to cell surface translocation. In support of the idea that stable expression of MR1 on the cell surface is tightly regulated, we observe that even the presence of intracellular infection with Mtb or incubation with soluble fractions from Mtb, there is little to no increase in the level of surface MR1 despite clear activation of MAIT cells. Similarly, translocation of MR1 to the cell surface in the context of bacterial ligands was not observed with soluble fractions from *E*. *coli* [29], suggesting a common regulatory pathway.

Mtb-derived MR1 ligands have not yet been identified, and we are not able to track the known small molecule ligands directly in cells. We show clear evidence, however, that trafficking of MR1 in endosomal compartments is key to Mtb antigen presentation, and hypothesize that the regulation of endosomal trafficking is key to generation of MR1^+^ EC that can encounter Mtb ligands and subsequently traffic to the cell surface. Further characterization of targets identified in this study will provide insight into the novel regulation of loading of ligand on MR1 and translocation of MR1 to the cell surface in the context of infection. The clear demonstration of endosomal trafficking as essential in the MR1-dependent activation of MAIT cells suggests a different paradigm for how MAIT cell recognition occurs and provides a critical first step in defining the fundamental mechanisms for MR1 antigen processing and presentation.

## Materials and Methods

### Bacteria and cells

*Mycobacterium tuberculosis* H37Rv (ATCC) or H37Rv-expressing dsRED, a kind gift from Joel Ernst was grown in Middlebrook 7H9 broth supplemented with Middlebrook ADC (Fisher), 0.05% Tween-80, and 0.5% glycerol. Before infection, bacteria were passaged 15 times through a tuberculin syringe to obtain a single cell suspension. For culture of colony forming units, bacteria were plated on 7H10 agar supplemented with Middlebrook ADC.

The bronchial epithelial cell line BEAS-2B (CRL-9609) was obtained from ATCC and cultured in DMEM + 10% heat inactivated FBS. NHBE cells were obtained from Lonza and were cultured in BEGM as recommended. CD8^+^ T cell clones D160 1–23, D466 H4, and D426 B1 have been previously described [3, 30, 31]. Briefly, D160 1–23 is HLA-E restricted, D466 H4 is HLA-B45 restricted, and D426 B1 is MR1 restricted. T cell clones were expanded as previously described [32].

### Ethics statement

This study was conducted according to the principles expressed in the Declaration of Helsinki. Study participants, protocols, and consent forms were approved by the Oregon Health & Science University Institutional Review Board (IRB00000186). Written informed consent was obtained from all participants. Uninfected adults were recruited from employees at Oregon Health & Science University as previously described [33]. Uninfected individuals were defined as healthy individuals with a negative tuberculin skin test and no known risk factors for infection with Mtb.

### Reagents and antibodies

Enzymes used for molecular cloning were purchased from New England Biolabs. The following antibodies or reagents were used for fluorescence microscopy and flow cytometry: α-MR1 (26.5, gift from Ted Hansen, biotinylated by Biolegend), α-Class I (W6/32, Serotec), α-TGN46 (Abcam), α-ERGIC-53 (Abcam), goat anti-mouse and anti-rabbit Alexafluor secondary antibodies (Life Technologies), α-β-2-microglobulin (SantaCruz) streptavidin-Alexa647 (Life Technologies), and CellLights reagents (Life Technologies). 6-formyl pterin was obtained from Schirck’s Chemical.

### Lentiviral shRNA library screen

A subgenomic lentiviral short hairpin RNA (shRNA) library targeting 114 genes trafficking genes was obtained in collaboration with L. Moita through the RNAi Consortium (Broad Institute). Genes were represented by at least five independent shRNA constructs arrayed in individual wells. The assay design is outlined in S1 Fig. Infection of BEAS-2B cells with lentivirus was performed as follows. BEAS-2B cells were plated on 96-well tissue culture plates at a concentration of 5 x 10^3^ cells per well in 100ul DMEM+10%FBS. After 24 hours the media was removed and replaced with 10ul virus and 40ul media containing 8ug/ml polybrene and the plate was centrifuged at 2200 rpm for 90 min at 37°C. After centrifugation, the media was replaced with 100ul fresh DMEM+10%FBS. The cells were incubated for 48 hours, then selected with 5ug/ml puromycin for an additional 72 hours before analysis by ELISPOT assay as follows. Treated cells were infected with H37Rv Mtb (MOI:30) for 18 hours. Following infection, cells were harvested and divided into four parts, one for counting and three for ELISPOT assays as described below. To count the relative number of cells per well, samples were fixed, combined with a fixed number of latex beads, and analyzed by flow cytometry. Wells that contained fewer than 20 cells per 10,000 latex beads were excluded from analysis as APCs from these wells did not elicit IFNγ response above background. The other three parts were plated as antigen presenting cells (APC) in IFNγ ELISPOT plates with MR1 (D426B1), HLA-E (D160 1–23), or HLA-B45 (D466H4)-restricted T cells clones. Each well was given a rank order based on the relative number of cells, which was plotted against the number of IFNγ Spot Forming Units (SFU). Each plate of shRNAs was assayed independently three times with each of the three T cell clones. Prism (GraphPad) was used for data analysis. Each well was given a rank value based on the relative number of cells and the rank was plotted in ascending order in relation to the number of spots per well. A linear regression analysis was performed to generate a best-fit line with 95% confidence intervals. Genes were considered candidates if two of the five independent shRNA-silenced wells resulted in T cell response that was reduced by at least 25% compared to the best-fit line in at least two of the three replicates. Selected candidates were confirmed using gene-specific siRNA as described below.

### siRNA knockdown

BEAS-2B cells were plated in 24-, 12-, or 6-well tissue culture plates (Corning), or 1.5mm glass-bottom chamber slides (Nunc) at 70% confluency and transfected with 50nM siRNA (Life Technologies) using HiPerfect (Qiagen). After 72 hours, cells were used in assays as indicated below.

### RNA isolation, cDNA synthesis, and real-time PCR analysis

Real-time PCR was used to analyze gene expression. Total RNA was isolated from samples treated with negative control or gene-specific siRNAs using the RNeasy Mini Kit following the manufacturer’s protocol (Qiagen). cDNA was synthesized from total RNA using the High Capacity cDNA Reverse Transcription Kit following the manufacturer’s protocol (Life Technologies). Real time PCR was carried out using TaqMan Universal PCR Master Mix (Life Technologies) on a Step One Plus Real-Time PCR System (Applied Biosystems). The FAM-MGB TaqMan Gene Expression Assays for all targets were obtained from Life Technologies. Reactions were run in triplicate in three independent experiments. Expression data were normalized to the housekeeping gene GAPDH and relative expression levels were determined using the 2^-ΔΔCT^ method described by Livak and Schmittgen [34].

### Generation and Expression of MR1-GFP

pCI MR1-eGFP was constructed using a cDNA ORF clone for MR1 isoform 1 purchased through Origene (RG220474, Ref seq: MN_001531.1) utilizing a Mega-primer PCR protocol. MR1 was first amplified by PCR so that an EcoRI site was integrated into the N-terminal end of the amplicon and a 25 bp overlap with the sequence for the following eGFP amplicon was added to the C-terminus (Primers: MR1_FW and MR1_RV). eGFP was then amplified out of a stock plasmid in our lab with a QGGGGFE flexible linker added to the N-terminus and a KpnI restriction site added to the C-terminal of the amplicon (Primers: QGGGGFE eGFP_FW and QGGGGFE eGFP_RV). The gel-purified MR1 and eGFP amplicons were then amplified in a PCR reaction using the MR1 amplicon as a mega-primer for the reaction. Following PCR purification (Qiagen cat. 28104) of the MR1 eGFP amplicon, an additional PCR reaction was run using primers initiating amplification from each terminal end of the template (MR1_FW and QGGGGFE eGFP_RV). Following gel purification (Qiagen cat. 28704), the 1.8kb MR1 eGFP insert was cloned into the pCI mammalian expression vector (Promega Cat. E1731) using EcoRI and KpnI restriction sites. Confirmatory sequencing of our construct revealed a missense mutation (A116G) within the Origene MR1 sequence that changed the coding for amino acid 39 from histidine to arginine. This mutation was repaired using mega-primer PCR utilizing a forward primer that amplified the N-terminal of pCI MR1 eGFP and a 30 bp reverse primer (MR1 A116wt_ RV) that spanned the region of the mutation and contained the correct A116 nucleotide. The resulting 145 bp amplicon was then used as a primer along with the reverse primer (QGGGGFE eGFP_RV) for a subsequent PCR reaction with pCI MR1 eGFP. The resulting PCR product was restriction cloned into pCI using EcoRI and KpnI sites. The completed plasmid was confirmed correct by sequencing through the entire MR1 eGFP insert.

N-terminal Tag-RFP Stx18 and VAMP4 fusion proteins were produced as complete genes in a production plasmid (Integrated DNA Technologies) containing EcoRI and KpnI restriction sites. The Tag-RFP gene was cut from the production plasmid, gel purified and subcloned into the pCI mammalian expression vector (Promega Cat. #E1731) using EcoRI and KpnI restriction sites.

BEAS-2B cells were transfected with the MR1-GFP, Stx18-RFP, or VAMP4-RFP plasmid constructs using Lipofectamine2000 (Life Technologies) or by nucleofection with Amaxa kit T (Lonza), program G-016. NHBE cells were transfected with plasmid constructs using the Amaxa nucleofector, with kit VPI-1005 (Lonza) and program W-001. For experiments with CellLights, reagent was added one hour after transfection with pCI:MR1-eGFP. Experiments with VAMP4-RFP co-transfection and Brefeldin A treatment were performed using BEAS-2B cells that were stably transduced with MR1-GFP lentiviral particles produced by The University of Pennsylvania Vector Core.

### ELISPOT assays

BEAS-2B cells in 12- or 6-well tissue culture plates were transfected with siRNAs as indicated above for 72 hours. siRNA transfected cells were infected with Mtb-dsRED (MOI:10 or as indicated) for 18 hours, then harvested, counted, and used in equivalent numbers as APC (5e3/well) in an IFNγ ELISPOT assay with 1e5 D426 B1, D160 1–23, and D466 H4 CD8^+^ T cell clones per well as previously described [31]. The data were compared using the student’s t-test, and all data presented are the average of a minimum of three independent replicates. For analysis of the functionality of MR1-GFP, BEAS-2B cells were transfected with MR1-GFP, or mock transfected. After 48 hours, cells were infected for 18 hours, then harvested, counted and plated at equivalent numbers in an IFNγ ELISPOT assay as described above. For the ELISPOT experiments with ‘Msm-sup’, *M*. *smegmatis* was cultured with shaking for 24 hours, then pelleted. The supernatant was passaged over a 0.22uM filter, then used directly in the ELISPOT assay.

### CFU assays

Equivalent numbers of cells treated with siRNA and infected with Mtb as indicated above were lysed and serial dilutions were plated on 7H9 culture plates. Colonies were enumerated after 12–14 days.

### MR1 surface stabilization assay

BEAS-2B cells plated in a 12-well tissue culture plate were transfected with siRNAs as indicated above for 72 hours. In the case of VAMP4, the cells were incubated with siRNA for 48 hours. After 72 (48) hours, cells were transfected with pCI:MR1-GFP and incubated for 24 hours. Cells were then incubated at 37°C with 50uM 6-formyl pterin for 16 hours. Cells were harvested on ice and surface stained with a primary antibody against MR1 (26.5, a kind gift from Ted Hansen, biotinylated, 1:100) for 40 min on ice in the presence of 2%HuS, 2% goat serum, and 0.5% FBS. After washing, streptavidin-Alexafluor 647 was added for 40 min on ice. Cells were washed and fixed, and subsequently analyzed with a BD FACSCanto II flow cytometer and FACS Diva software (BD). All analyses were performed using FlowJo software (TreeStar).

### Fluorescence microscopy

BEAS-2B cells in 1.5mm glass bottom chamber slides (Nunc) were transfected with pCI:MR1-GFP (2ug/1e6 cells). Transfected cells were infected with H37Rv-dsRED, or treated with ligands as indicated above. Cells were washed, fixed with 1% paraformaldehyde for 18 hours, then stained as indicated above with the 26.5 antibody. Images were acquired on a high-resolution wide-field CoreDV system (Applied Precision) with a Nikon Coolsnap ES2 HQ. Each image was acquired as z-stacks in a 1–24x1024 format with a 60x 1.42 NA Plan Apo N objective. Images were deconvolved with an optical transfer function using an iterative algorithm of 10 iterations. Acquired images were analyzed using Imaris (Bitplane). Analysis of MR1-eGFP EC colocalization with CellLights reagents or antibody staining was performed using the “Spots” module of Imaris and the “spots colocalization” MatLab Xtension module as described in S3 Fig.

### Data analysis

Data were analyzed and plotted using Prism 5 (GraphPad Software). Statistical significance was determined using Student’s two-tailed t test, unless otherwise indicated.

## Supporting Information

S1 Movie MR1-GFP localizes to endosomal compartments. BEAS-2B cells were transfected with pCI:MR1-GFP and incubated for 30 hours. Cells were imaged live every 5 seconds for 10 minutes.
(MOV)

S2 Movie MR1-GFP localizes to endosomal compartments with late endosome and lysosome markers. BEAS-2B cells were transfected with pCI:MR1-GFP and incubated for 30 hours. One hour after transfection, cells were incubated with the lysosome Organelle light reagent. Cells were imaged live every 5 seconds for 10 minutes.
(MOV)

S1 Fig shRNA library screen workflow. BEAS-2B cells were seeded in 96-well tissue culture plates at 5,000 cell per well and incubated for 18 hours. Cells were infected with lentivirus in the presence of polybrene by spinoculation for 90 minutes at 37°C. The lentiviral library was arrayed such that each gene in the library was represented by at least 5 unique lentiviral shRNA constructs in individual wells. Infected cells were incubated for 48 hours, then selected with 5ug/ml puromycin for an additional 48 hours. Cells were then infected for 18 hours with Mtb-dsRED (MOI:30). Infected cells were harvested and used as antigen presenting cells (APC) in an IFNγ ELISPOT assay with MR1-, HLA-E, and HLA-B45 T cell clones in parallel. A subset of the APC from each well was fixed with 1% PFA, then spiked with latex beads and analyzed by flow cytometry to determine the relative number of cells harvested from each well. Prism (GraphPad) was used to plot the number of IFNγ ELISPOTs from each well versus the relative number of cells per well, and to generate a regression line with a 95% confidence interval. Each shRNA was assayed in triplicate with each of the three T cell clones. The response of the T cell clones was analyzed and wells were considered hits if the response was at least 25% below the regression line for at least two of the three replicates. Genes were considered putative candidates if at least two of the five independent shRNA constructs met the threshold for a hit.
(TIF)

S2 Fig Impact of gene knock down on trans-Golgi network (TGN) structure. BEAS-2B cells were treated with missense, Stx18, Rab6, or VAMP4 siRNA for 72 hours. Cells were fixed, stained with α-TGN46, and imaged. Shown are representative images from three independent experiments.
(TIF)

S3 Fig Quantification of endosomal compartments using Imaris. BEAS-2B cells were transfected with pCI-:MR1-GFP and co-incubated with RFP CellLights reagents for lysosomes (Lamp1) for 48 hours, then imaged live. The top row displays individual images and a merge of a representative cell expressing MR1-GFP and Lamp1-RFP. MR1-GFP^+^ and Lamp1^+^ EC were quantified using the “Spots” function on Imaris as shown on the bottom left. Lamp1^+^ EC co-localizing with MR1-GFP^+^ EC were identified using the “Spots colocalization” MatLab Xtension module of Imaris. Quantification of the total number of MR1-GFP^+^ EC and the number of MR1-GFP^+^ Lamp1^+^ EC for the representative cell is shown in the graph on the bottom right. This analysis was repeated for each cell imaged and the average number of dual positive endosomes for all cells imaged in graphed in Fig 1D.
(TIF)
