# Supplementary figures and images for: Endosomal MR1 Trafficking Plays a Key Role in Presentation of Mycobacterium tuberculosis Ligands to MAIT Cells
Source: PLoS Pathog. 2016 Mar 31;12(3):e1005524. doi: 10.1371/journal.ppat.1005524 (PMC4816560; doi:10.1371/journal.ppat.1005524)

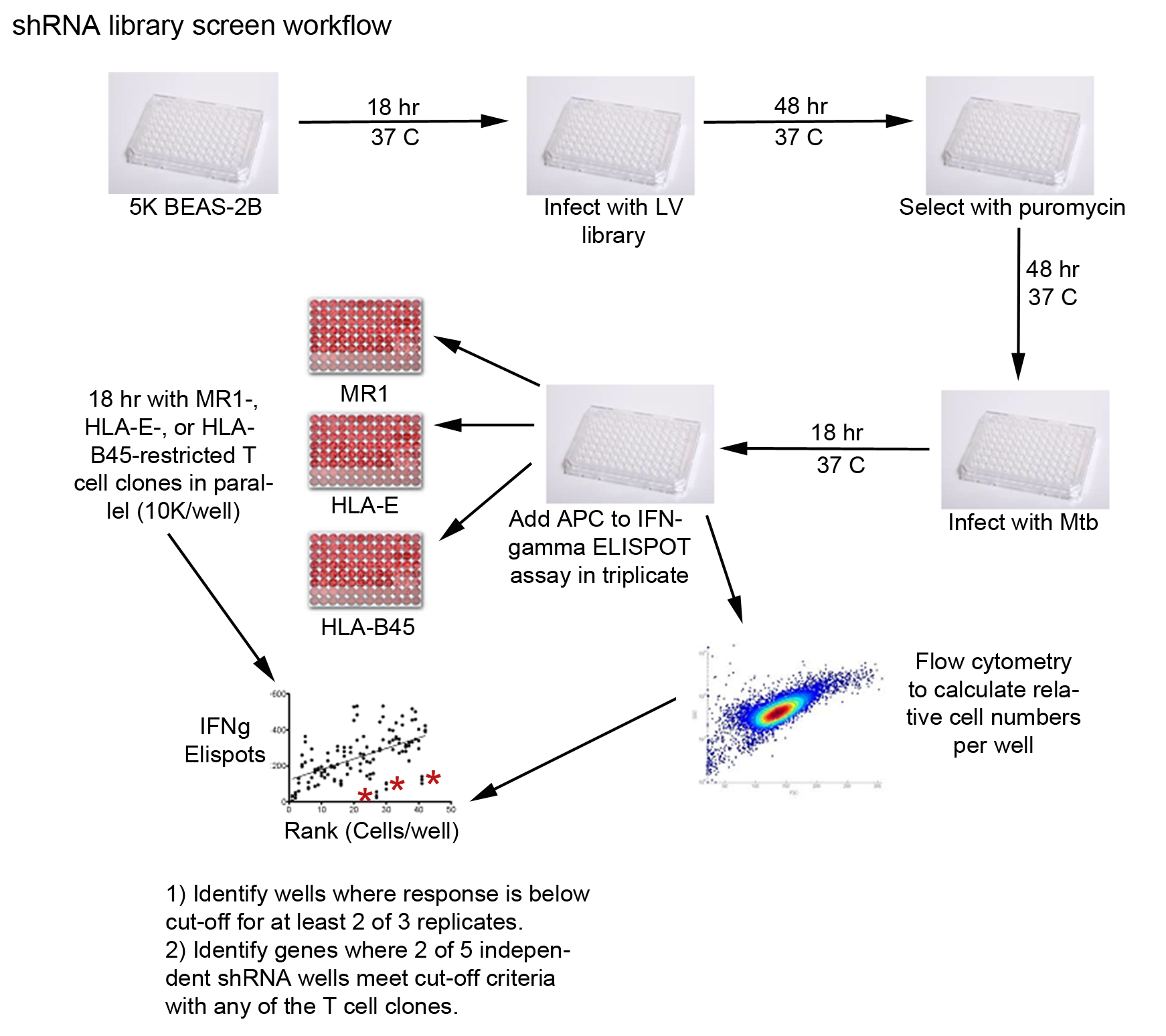

Supplement: S1 Fig — BEAS-2B cells were seeded in 96-well tissue culture plates at 5,000 cell per well and incubated for 18 hours. Cells were infected with lentivirus in the presence of polybrene by spinoculation for 90 minutes at 37°C. The lentiviral library was arrayed such that each gene in the library was represented by at least 5 unique lentiviral shRNA constructs in individual wells. Infected cells were incubated for 48 hours, then selected with 5ug/ml puromycin for an additional 48 hours. Cells were then infected for 18 hours with Mtb-dsRED (MOI:30). Infected cells were harvested and used as antigen presenting cells (APC) in an IFNγ ELISPOT assay with MR1-, HLA-E, and HLA-B45 T cell clones in parallel. A subset of the APC from each well was fixed with 1% PFA, then spiked with latex beads and analyzed by flow cytometry to determine the relative number of cells harvested from each well. Prism (GraphPad) was used to plot the number of IFNγ ELISPOTs from each well versus the relative number of cells per well, and to generate a regression line with a 95% confidence interval. Each shRNA was assayed in triplicate with each of the three T cell clones. The response of the T cell clones was analyzed and wells were considered hits if the response was at least 25% below the regression line for at least two of the three replicates. Genes were considered putative candidates if at least two of the five independent shRNA constructs met the threshold for a hit. (TIF) [file ppat.1005524.s003.tif]

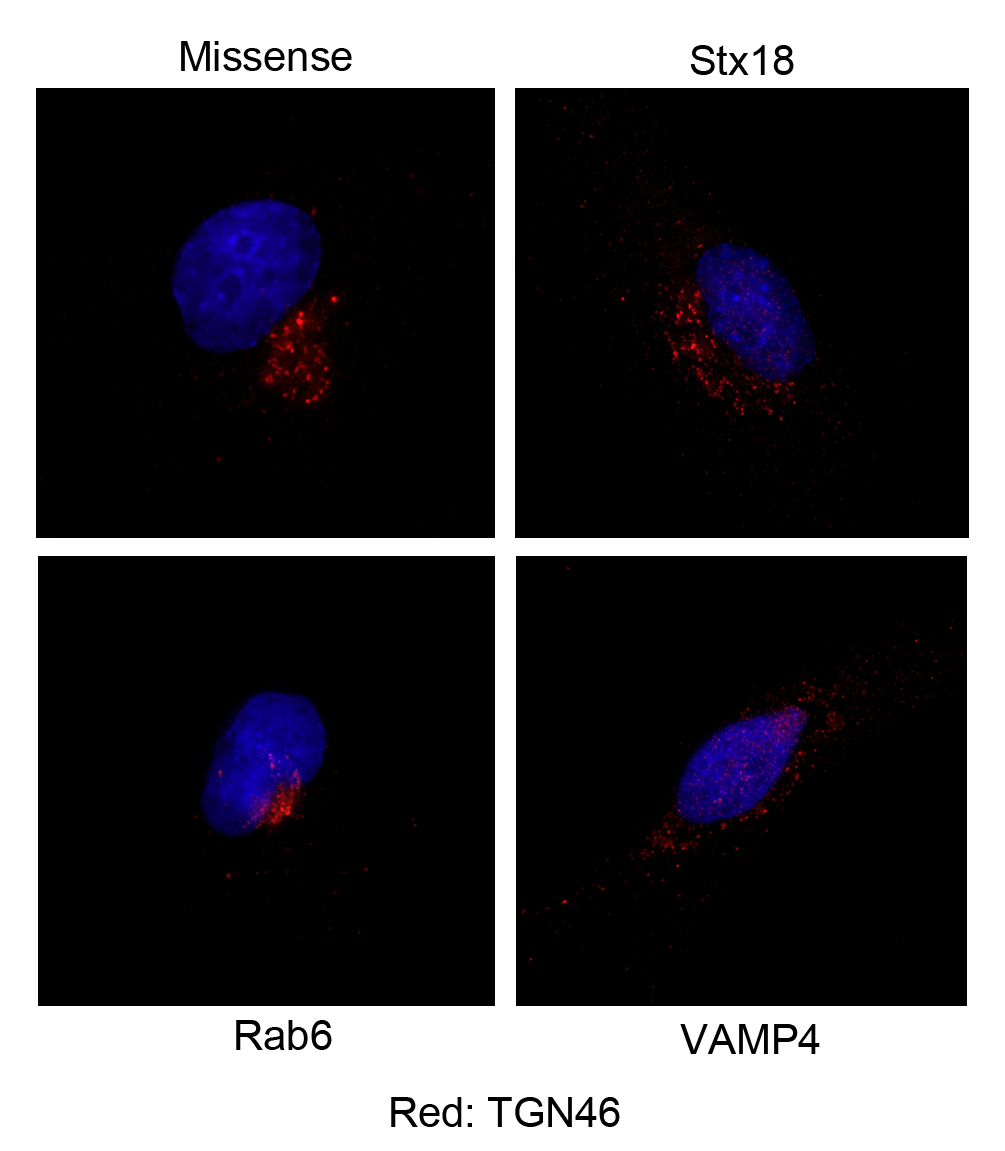

Supplement: S2 Fig — BEAS-2B cells were treated with missense, Stx18, Rab6, or VAMP4 siRNA for 72 hours. Cells were fixed, stained with α-TGN46, and imaged. Shown are representative images from three independent experiments. (TIF) [file ppat.1005524.s004.tif]

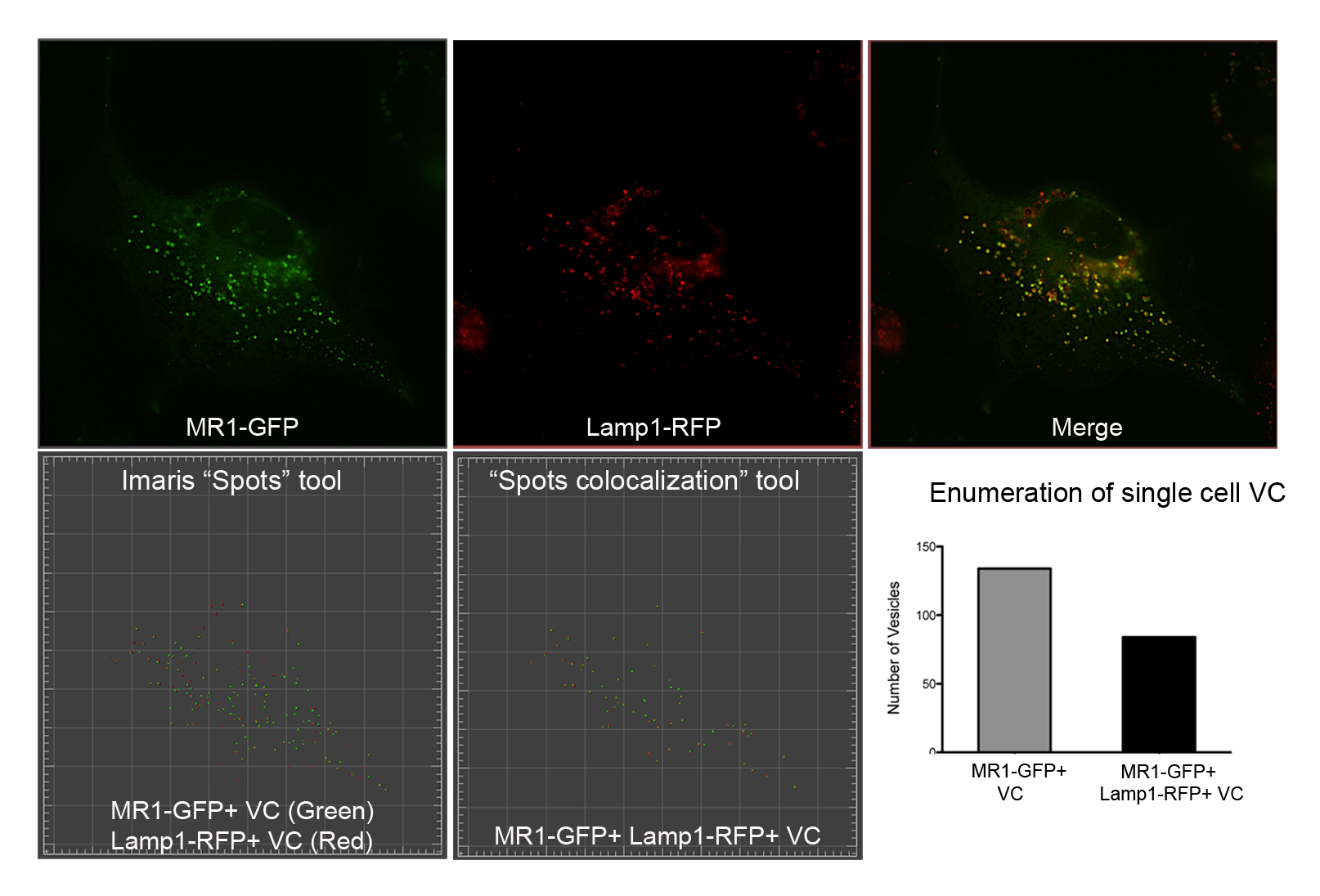

Supplement: S3 Fig — BEAS-2B cells were transfected with pCI-:MR1-GFP and co-incubated with RFP CellLights reagents for lysosomes (Lamp1) for 48 hours, then imaged live. The top row displays individual images and a merge of a representative cell expressing MR1-GFP and Lamp1-RFP. MR1-GFP+ and Lamp1+ EC were quantified using the “Spots” function on Imaris as shown on the bottom left. Lamp1+ EC co-localizing with MR1-GFP+ EC were identified using the “Spots colocalization” MatLab Xtension module of Imaris. Quantification of the total number of MR1-GFP+ EC and the number of MR1-GFP+ Lamp1+ EC for the representative cell is shown in the graph on the bottom right. This analysis was repeated for each cell imaged and the average number of dual positive endosomes for all cells imaged in graphed in Fig 1D. (TIF) [file ppat.1005524.s005.tif]
